# Molecular Insights into the Anticancer Activity of Withaferin-A: The Inhibition of Survivin Signaling

**DOI:** 10.3390/cancers16173090

**Published:** 2024-09-05

**Authors:** Renu Wadhwa, Jia Wang, Seyad Shefrin, Huayue Zhang, Durai Sundar, Sunil C. Kaul

**Affiliations:** 1 AIST-INDIA DAILAB, National Institute of Advanced Industrial Science & Technology (AIST), Central 4-1, Tsukuba 305-8565, Japan; renu-wadhwa@aist.go.jp (R.W.); wangjia@szbl.ac.cn (J.W.); s2130297@u.tsukuba.ac.jp (H.Z.); 2 Department of Biochemical Engineering & Biotechnology, Indian Institute of Technology (IIT) Delhi, Hauz Khas, New Delhi 110-016, India; bez188440@dbeb.iitd.ac.in; 3 Institute of Bioinformatics and Applied Biotechnology (IBAB), Bengaluru 560-100, India

**Keywords:** Withaferin-rich extract from Ashwagandha leaves (Wi-AREAL), Withaferin-A, Withanone, cancer, EMT inhibition, Survivin signaling, therapy

## Abstract

**Simple Summary:**

The inactivation of apoptotic signaling by inhibitors of apoptosis proteins (IAPs) is one of the important hallmarks of cancer cells. Survivin, a 16.5 kDa member of the IAP family, is commonly enriched in many cancers and is a potential therapeutic target. We investigated the Survivin-targeting potential of Withaferin-A (Wi-A) and Withanone (Wi-N), two major withanolides from *Withania somnifera* (Ashwagandha), using computational assays. The results were validated in various in vitro experimental assays using Wi-A-rich extracts from Ashwagandha leaves, suggesting their use as an important bioresource for cancer drug development.

**Abstract:**

Survivin, a member of the IAP family, functions as a homodimer and inhibits caspases, the key enzymes involved in apoptosis. Several Survivin inhibitors, including YM-155, Debio1143, EM1421, LQZ-7I, and TL32711, have emerged as potential anticancer drugs awaiting validation in clinical trials. Due to the high cost and adverse side effects of synthetic drugs, natural compounds with similar activity have also been in demand. In this study, we conducted molecular docking assays to evaluate the ability of Wi-A and Wi-N to block Survivin dimerization. We found that Wi-A, but not Wi-N, can bind to and prevent the homodimerization of Survivin, similar to YM-155. Therefore, we prepared a Wi-A-rich extract from Ashwagandha leaves (Wi-AREAL). Experimental analyses of human cervical carcinoma cells (HeLa and ME-180) treated with Wi-AREAL (0.05–0.1%) included assessments of viability, apoptosis, cell cycle, migration, invasion, and the expression levels (mRNA and protein) of molecular markers associated with these phenotypes. We found that Wi-AREAL led to growth arrest mediated by the upregulation of p21^WAF1^ and the downregulation of several proteins (CDK1, Cyclin B, pRb) involved in cell cycle progression. Furthermore, Wi-AREAL treatment activated apoptosis signaling, as evidenced by reduced PARP-1 and Bcl-2 levels, increased procaspase-3, and elevated Cytochrome C. Additionally, treating cells with a nontoxic low concentration (0.01%) of Wi-AREAL inhibited migration and invasion, as well as EMT (epithelial–mesenchymal transition) signaling. By combining computational and experimental approaches, we demonstrate the potential of Wi-A and Wi-AREAL as natural inhibitors of Survivin, which may be helpful in cancer treatment.

## 1. Introduction

Survivin/BIRC5 is the smallest (16.5 kDa, 142 amino acids) member of the IAP (Inhibitor of apoptosis protein) family, which contains eight members (BIRC1 to BIRC8) possessing a ~70 amino acid long baculoviral IAP repeat (BIR) domain. Human Survivin harbors the BIR domain (18–88 aa) in the N-terminal, a linker (89–102 aa), and an extended α-helix (98–142 aa) in the C-terminal. Protein homodimers and their BIR domains are stabilized by interactions of 6–10 N-terminal residues with a linker region and a zinc finger (Cys57, Cys60, His77, and Cys84), respectively [1]. These contain cell-cycle-dependent elements and a cell cycle gene homology region in its promoter, which is regulated by various cell survival signaling cascades (including receptor tyrosine kinases of EGFR, IGF-1R, and HER2 and their downstream effector pathways of PI3K/Akt/mTOR, MEK/MAPK, STAT3, and HIF-1α). Enriched at the G2/M phase of the cell cycle [2], the Survivin homodimer (stabilized by phosphorylation at Thr34) has been shown to bind directly to (i) cell death proteases caspase-3 and -7, inhibiting their caspase activity and apoptosis in response to a diverse range of stimuli, and (ii) microtubules of the mitotic spindle, regulating cytokinesis [2,3,4]. Overexpressed in many forms of cancer, Survivin has been shown to promote cancer cell proliferation, cell cycle progression, angiogenesis, and therapeutic resistance [2,4,5]. Most recently, it has been shown to be a dynamic multitasking protein in the cytoplasm, mitochondria, nucleus, exosomes, cell membrane, and extracellular matrix [1,6]. Due to its prominent role in three major cancer phenotypes (cell survival, metastasis, and drug resistance) and lack of expression in most adult normal tissues, it is considered an important cancer drug target that regulates autophagy and cancer cell stemness [1,7,8,9].

Transcriptional (ribozymes, antisense oligonucleotide drugs, siRNAs, and miRNAs) and post-transcriptional (CDK inhibitors to block Thr34 phosphorylation and promote its destabilization and degradation) small-molecule inhibitors of Survivin have been reported [1,10,11]. Broadly, these include molecules (i) causing the downregulation of transcription, (ii) promoting mRNA degradation, (iii) abrogating protein homodimerization, and (iv) attenuating interactions with partner proteins, as well as (v) specific peptides and antibodies [11]. The downregulation of Survivin expression/function has been shown to sensitize tumor cells to a variety of chemotherapeutic drugs, including cisplatin, paclitaxel, STX140, STX641, and trichostatin A [6,12,13,14,15,16,17,18], and inhibit EMT (epithelial–mesenchymal transition) [11,19]. The inhibition of Survivin dimerization by small molecules was shown to induce its degradation in a proteasome-dependent manner [20]. Among several small-molecule inhibitors of Survivin, including YM-155, HY-10194, Debio1143, EM1421, FL118, Flavokawain A, GDP366, LQZ-7I, LQZ-7F, and TL32711, emerging from preclinical studies, YM-155 (sepantronium bromide) has been extensively studied and shown to inhibit Survivin expression (at both the mRNA and protein levels with high potency and specificity) and tumor growth in a variety of human cancer xenograft models [21,22,23,24,25]. Furthermore, it was shown to revert radio resistance [22] and activate drug-induced apoptosis [22,26]. However, clinical trials of YM-155 were limited by its maximum tolerated dose [1] and off-target effects [27]. On the other hand, the anticancer activity of some herbal extracts is mediated by the downregulation of Survivin and subsequent activation of apoptosis-promoting proteins, including caspase 3 and PARP-1 [28,29,30].

Ashwagandha leaf extract and its bioactive withanolides (Withaferin-A, Wi-A, and Withanone, Wi-N; Supplementary Figure S1A) have been shown to possess remarkable multi-model anticancer activities [29,30,31,32,33,34,35,36,37,38]. Compared to Wi-N, Wi-A showed higher potency, whereas the former showed higher selectivity toward cancer cells [31]. Wi-N was shown to possess a strong binding affinity to the BIR5 domain of Survivin and interfere with its inhibitory action against caspases and apoptosis [39]. Zhou et al. [40] and Chang et al. [41] have shown that targeting Survivin results in sensitizing cervical cancer cells to radio and chemotherapeutic treatments. However, there is only limited information on Survivin-targeting natural compounds. In this premise, we first investigated the Survivin-inhibitory potential of Wi-A and Wi-N concerning YM-155 using a computational approach and validated the findings by in vitro experiments treating cervical cancer cells (HeLa and ME-180) with Wi-A-rich Ashwagandha leaf (Wi-AREAL) extract.

## 2. Material and Methods

### 2.1. Computational Techniques

#### 2.1.1. Docking of Withaferin-A, Withanone, and YM-155 with Survivin

The crystal structure of the Survivin homodimer was obtained from the PDB data repository “https://www.rcsb.org/structure/3uih (accessed on 23 January 2023)” using PDB ID: 3UIH [42]. The Wi-A, Wi-N, and YM-155 (known inhibitor) ligand structures were obtained from the PubChem repository using CID:265237, CID:2169027, and CID:10126189. The protein and ligand structures were pre-processed using the Protein Preparation wizard and Ligprep module in the Schrodinger Maestro suite [43]. A docking grid was generated to focus on the Survivin homodimer-interacting region from residue Phe93 to Lys102 [44]. The extra-precision algorithm of the Glide docking program was then utilized to perform flexible docking [45].

#### 2.1.2. All-Atom Molecular Dynamics Simulations of the Ligand–Survivin Complex

The stability of the interaction between Wi-A and YM-155 with Survivin was assessed through MD simulation using the Desmond module of Schrodinger software version 2020-1 [46,47]. The docked protein–ligand complexes were solvated with a TIP4P water model using Desmond’s ‘system builder’ program. An orthorhombic periodic boundary condition box was set at 10 Å, and ions like (Na+/Cl−) were added to both systems to neutralize the overall charge of the system. The systems were then energy minimized using small steps of Brownian dynamics for 20 ps at a low temperature of 10 K in the NVT ensemble to avoid steric hindrance [48]. After energy minimization, the systems were equilibrated in seven steps in both the NVT and NPT ensembles using the relaxation protocol option available in the Desmond package. The production simulations were conducted in the NPT ensemble for 300 ns with a time step of 2 fs while keeping the pressure and temperature of the systems at one atmospheric pressure and 300 K temperature, respectively. The Nose–Hoover chain thermostat and Martyna–Tobias–Kelin barostat were used in the temperature and pressure coupling simulations, respectively. The cut-off radius for short-range Coulomb interactions was set to 9 Å, and no molecules were restrained during the simulation.

#### 2.1.3. Analyzing the Simulation Trajectory

The Schrodinger suite’s Simulation Event Analysis module analyzed the simulation trajectory [44]. The stability of the protein–ligand complexes was investigated over time using root mean square deviation (RMSD). The number of hydrogen bonds and their occupancy percentage over the simulations formed between the ligand and protein were calculated. The flexibility, binding ability inside the active pocket of protein, and stability of the ligands over the simulation period were analyzed using the RMSD [49]. Finally, the PyMOL suite of the Schrodinger package was used to visualize the interaction between the ligand and the protein structure (The PyMOL Molecular Graphics System, Version 2.0 Schrödinger, LLC, New York, NY, USA).

#### 2.1.4. MM/GBSA Binding Free Energy

The Schrodinger suite’s ‘Average Structure’ module chose the average representative structure from the simulation trajectories. The Schrodinger suite’s Prime MM/GBSA module was utilized to calculate the binding free energy [50]. The equation used for the calculation was MM/GBSA ∆G bind = ∆G complex − (∆G receptor + ∆G ligand), where ∆G complex, ∆G receptor, and ∆G ligand represent the free energies of the complex, receptor, and ligand, respectively. MM/GBSA refers to the binding energy, with a more negative value indicating a higher ligand affinity toward the target protein [51]. However, the calculated binding energy values were not absolute but relative. This is due to the inherent limitation of end-point energy calculation methods.

### 2.2. Experimental Techniques

#### 2.2.1. Cell Culture and Treatments

Human normal lung fibroblasts (MRC-5, TIG-3, and WI-38) and a variety of cancer cells, including colon cancer (HCT116), breast cancer (MDA-MB-231, MCF-7 and T-47D), fibrosarcoma (HT1080), non-small lung cancer (A549), cervical cancer (HeLa, ME-180, SKG-II, and CaSki), osteosarcoma (U2OS and Saos-2), and melanoma (G361), were purchased from the Japanese Collection of Research Bioresources (JCRB, Tokyo, Japan). Cells were cultured in Dulbecco’s modified Eagle’s medium (DMEM, Wako, Osaka, Japan) supplemented with 10% (*v*/*v*) fetal bovine serum (FBS, Wako, Osaka, Japan) and 1% penicillin/streptomycin in a humidified incubator (37 °C and 5% CO_2_). A Withaferin-A (Wi-A)-rich extract of Ashwagandha leaves (Wi-AREAL) was prepared as described earlier [52]. The purified compounds Wi-A (C_28_H_38_O_6_, steroidal lactone) and YM-155 (1-(2-methoxyethyl)-2-methyl-4,9-dioxo-3-(pyrazin-2-ylmethyl)-4,9-dihydro-1H-naphtho [2,3-d]imidazol-3-ium bromide) were obtained from Sigma-Aldrich, St. Louis, MO, USA, and MedChemExpress, Monmouth Junction, NJ, USA, respectively. The structures of the compounds are shown in Supplementary Figure S1A. They were dissolved in DMSO (Wako, Osaka, Japan) to create 5 mM stocks. DMSO was used as the solvent control in all experiments. Cells were treated with the test reagents diluted in the cell culture medium to achieve the indicated working concentration.

#### 2.2.2. HPLC Analysis

The concentration of Wi-A and Wi-N in the Wi-AREAL were estimated by reversed-phase HPLC using Develosil C30-UG Column (Batch No. 030718; Nomura Chemical Co., LTD, Seto, Aichi, Japan) on a Shimadzu HPLC CBM-20A/20Alite (Shimadzu Corporation, Tokyo, Japan) equipped with a UV detector (SPD-20A/20AV). The separation was carried out at a 1 mL/min flow rate at a column temperature of 40 °C. Gradient extraction was performed with water (Solution A) and methanol (Solution B). The 30 min gradient program (Solution B—50% for 0.01 min, 50–80% for 25 min, and 50% for 5 min before stopping the pump) was applied. The detection of components in eluted fractions was carried out at 237 nm.

#### 2.2.3. Cell Viability Assay

The cytotoxicity of the test reagents was assessed using an MTT (3-(4,5-dimethylthiazol-2-yl)-2,5-diphenyltetrazolium bromide) assay as previously described [52]. Cells were seeded in a 96-well plate and incubated at 37 °C overnight. After the cells adhered well, they were treated with Wi-AREAL and YM-155 (for either 24 or 48 h, as indicated in the Section 3), followed by the addition of MTT (10 μL of 5 mg/mL to each well; Sigma-Aldrich, St. Louis, MO, USA). The plates were then incubated at 37 °C for 4 h, after which DMSO (100 µL per well) was added, and the plates were shaken for 15–20 min. The optical density was measured at 570 nm using a Tecan Infinite Pro microplate reader (Tecan Group Ltd., Männedorf, Switzerland).

#### 2.2.4. Morphological Observations

Cells were seeded (2 × 10^5^ cells/well in 6-well plates) and allowed to settle overnight, followed by incubation with different concentrations of Wi-AREAL for 24–48 h. The morphology of cells was observed, and the images were captured using a phase-contrast microscope (Nikon Eclipse TE300; Nikon, Tokyo, Japan).

#### 2.2.5. Colony Formation Assay

The effect of Wi-AREAL on long-term cell proliferation was determined by a colony-forming assay. Cells (500 per well) were seeded in 6-well plates and allowed to adhere to the surface overnight. They were then treated with DMEM containing Wi-AREAL for approximately 10 h. The cells were then maintained in a normal medium with regular medium changes every third day for 10–15 days. The experiment was concluded once the control plate surface was covered (~60–70%) with colonies. The plates were washed with cold PBS, and the colonies were fixed with pre-chilled methanol and acetone (1:1, *v*/*v*). Subsequently, they were stained with 0.1% crystal violet solution (Wako, Osaka, Japan) at room temperature overnight and then destained with water. The plates were left open for air drying, and images were captured by a scanner (Epson GTS640, Tokyo, Japan). Finally, the colonies were manually counted, and the results were plotted as a percent change with respect to the control.

#### 2.2.6. Flow Cytometry Analysis

Control and treated cells were harvested with 0.25% trypsin. Cell pellets were washed with cold PBS and then added drop-wise to pre-chilled 70% ethanol. The fixed cells were centrifuged at 500× *g* for 5 min at 4 °C, washed with cold PBS twice, and re-suspended in 1 mL cold PBS. The cell suspensions were treated with RNase A (100 μg/mL) at 37 °C for 1 h to avoid false DNA-PI staining, followed by brief centrifugation to discard the supernatant. Cells were re-suspended in 200 μL of Guava Cell Cycle reagent (Millipore, Billerica, MA, USA), incubated in the dark at room temperature for 30 min, and then subjected to Guava PCA flow cytometer (Millipore, Billerica, MA, USA). The data were further analyzed using FlowJo software (version 7.6, Ashland, OR, USA).

#### 2.2.7. Apoptosis Assay

Control and treated cells were collected by centrifugation at 1200 rpm for 5 min at 4 °C. Cells were re-suspended in a medium to make the concentration equal to 1 × 10^6^ cells/mL. Then, 100 μL of cell suspension from each group was incubated with 100 μL of Guava Nexin Reagent (Millipore, Billerica, MA, USA) at room temperature in the dark for 20 min and analyzed using a Guava PCA flow cytometer (Millipore). Apoptotic cells were determined using FlowJo software (version 7.6; LLC, USA).

#### 2.2.8. Western Blotting

Control and treated cells were lysed in RIPA buffer (Thermo Fisher Scientific, Waltham, MA, USA) supplemented with a complete protease inhibitor cocktail (Roche Applied Science, Mannheim, Germany). The concentrations of proteins in lysates were determined using the Pierce BCA Protein Assay Kit (Thermo Fisher Scientific, Waltham, MA, USA). The lysates containing equal amounts (10–20 μg) of protein were subjected to SDS-polyacrylamide gel electrophoresis followed by transfer to a polyvinylidene difluoride (PVDF) membrane (Millipore, Billerica, MA, USA) using a semidry transfer blotter (ATTO Corporation, Tokyo, Japan). Membranes were blocked with 3% bovine serum albumin (BSA; WAKO, Osaka, Japan) at room temperature for 1 h, followed by incubation with target protein-specific primary antibodies (summarized in Supplementary Table S1) at 4 °C overnight. The blots were incubated with secondary antibodies conjugated to horseradish peroxidase and detected by enhanced chemiluminescence reaction (ECL) (GE Healthcare, Amersham, Buckinghamshire, UK). The β-actin antibody (643807, BioLegend, Tokyo, Japan) was used as an internal loading control. Protein expression was quantified using ImageJ 1.45 software (National Institute of Health, Bethesda, MD, USA).

#### 2.2.9. Immunocytochemistry

Cells were seeded on 18 mm glass coverslips placed in a 12-well culture dish. Well-adhered cells were washed with cold PBS, fixed with pre-chilled methanol/acetone (1:1) for 10 min, permeabilized by incubation with 0.1% Triton X-100 in PBS (PBST) for 10 min, blocked with 2% bovine serum albumin in PBST for 1 h, and then incubated with anti-Survivin (71G4B7) antibody at 4 °C overnight. They were incubated with Alexa-594-conjugated goat anti-rabbit (Molecular Probes, Eugene, OR, USA) secondary antibody after washing (thrice) with PBST. The nucleus was stained with Hoechst 33342 (Invitrogen, Molecular Probes, Eugene, OR, USA) in the dark for 10 min. After extensive washing with PBST, the cells were mounted and visualized under a Carl Zeiss microscope (Axiovert 200 M; Tokyo, Japan). The images were quantitated using ImageJ software (National Institute of Health, Bethesda, MD, USA).

#### 2.2.10. RNA Extraction and Quantitative Real-Time Polymerase Chain Reaction

Total RNA was isolated and quantitated from control and treated cells using the RNeasy Mini Kit (Qiagen, Stanford Valencia, CA, USA) and NanoDrop ND-1000 (Nanodrop Technologies, Wilmington, DE, USA), respectively. An equal amount of RNA (1 μg) from samples was used for reverse transcription following the instructions of the QuantiTect Reverse Transcription Kit (Qiagen, Tokyo, Japan). Quantitative real-time polymerase chain reaction (PCR) was performed in triplicate using SYBR Select Master Mix (Applied Biosystems, CA, USA) on the Eco™ real-time system (Illumina, San Diego, CA, USA) with the following cycling conditions: 50 °C for 2 min, 95 °C for 10 min, and 40 cycles of denaturing at 95 °C for 15 s, annealing at 60 °C for 1 min, and extension at 72 °C for 15 s. To rule out technical inconsistency, the abundantly expressed housekeeping gene 18S was used as the internal control for each experiment. The results of the real-time qRT-PCR were analyzed and expressed as a relative expression of threshold cycle value, which was then converted to x-fold changes (2^−∆∆Ct^) in line with the manufacturer’s instructions. The sequences of primers used are listed in Supplementary Table S2.

#### 2.2.11. Wound Scratch Assay

Monolayers of cells in 6-well plates (at ~24 h after plating) were wounded by creating a uniform scratch on the surface using a 200 µL pipette tip. The cells were washed three times with PBS and then cultured in either the control culture medium or test compounds (Wi-AREAL at 0.01%, Wi-A at 0.5 µM, and YM-155 at 40 nM) as indicated. The movement of cells within the scratched area was observed and captured under a phase-contrast microscope using a 10X phase objective (Nikon Eclipse TE300; Nikon, Tokyo, Japan) at 0, 24, and 48 h. The experiment was repeated three times.

#### 2.2.12. Cell Invasion Assay

A cell invasion assay was conducted using the Corning BioCoat Matrigel Invasion Chamber (product number 354480; Corning, Labware, Inc., Two Oak Park, MA, USA) following the manufacturer’s instructions. To start, 750 μL of 10% fetal bovine serum-supplemented DMEM was added to a 24-well plate as a chemoattractant. Then, the control and 0.01% Wi-AREAL-treated cells suspended in 500 μL of serum-free DMEM medium were added into the invasion inserts. After 24 h of incubation, the non-invading cells were removed from the upper surface of the membrane by gentle scrubbing. Then, the cells were fixed and stained. Excessive stain was removed by rinsing with distilled water. The inserts were air-dried, visualized, and photographed under a phase-contrast microscope (Nikon Eclipse TE300; Nikon, Tokyo, Japan).

#### 2.2.13. Statistical Analysis

Data from three or more experiments were presented as mean ± standard deviation (SD). An unpaired *t*-test (GraphPad Prism 7.0, San Diego, CA, USA) was performed to determine the degree of significance between the control and experimental samples. Significant values were represented as * *p* < 0.05, ** *p* < 0.01, and *** *p* < 0.001.

## 3. Results

### 3.1. Withaferin-A Showed Good Docking to the Survivin Homodimer

**Figure 1:**
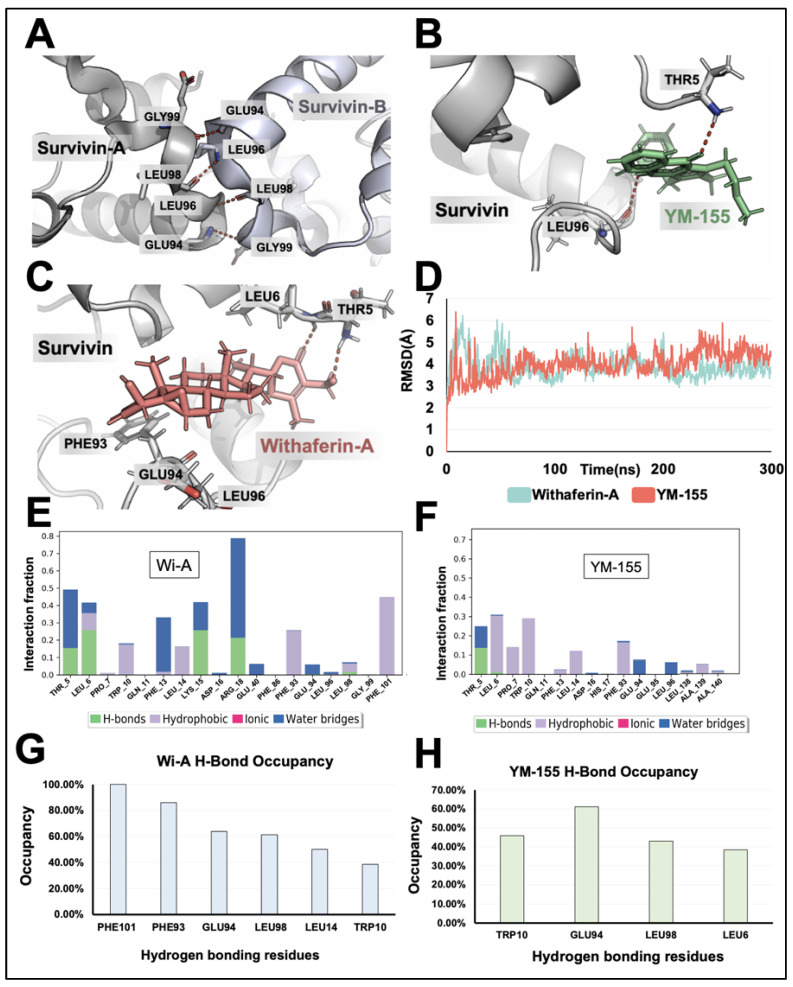
Computational analysis reveals Survivin inhibition by Withaferin-A and YM-155. (**A**) Visualization of the interaction between homodimers of Survivin involving amino acid residues Gly99, Leu98, Leu96, and Glu94. (**B**) Visualization of the interaction between YM-155 and the Survivin homodimer-forming region; the interaction of YM-155 with Thr5 and Leu96 is seen. (**C**) Visualization of the interaction between Wi-A and the Survivin homodimer-forming region; the interaction of Wi-A with Thr5 is seen. (**D**) Root means square deviation plot of the simulations for YM-155 and Wi-A interactions with Survivin. (**E**,**F**) Interaction fraction diagrams of Wi-A (**E**) and YM-155 (**F**) over the simulation were obtained using Schrodinger simulation analysis. (**G**,**H**) Hydrogen bond occupancy plot of Wi-A (**G**) and YM-155 (**H**) with Survivin obtained using VMD analysis.

Since the homodimer formation of Survivin is essential for its stability and function, we examined the interaction of Wi-A and Wi-N with the homodimer of Survivin in reference to YM-155. The crystal structure of Survivin revealed that the residues Glu94, Leu96, Leu98, and Gly99 were involved in the hydrogen bonding of the molecules in the Survivin homodimer (Figure 1A). Wi-A and YM-155 interacted within the homodimer-forming region with a docking score of −1.88 kcal/mol and −1.76 kcal/mol, respectively. Docking scores represent empirical values for the relative assessment of ligand binding; a higher docking score signifies more vital interaction. Furthermore, the ligands complexed with Survivin were simulated for 300 ns to determine the stability of the ligands in the binding region. From the average structure obtained from the simulation, it was apparent that YM-155 interacted within the homodimer-forming region, which also includes hydrogen bonding interactions with Leu96 and Thr5 (Figure 1B). From the visualization of the target, it was evident that Leu96 is one of the key residues holding the individual chains of Survivin together through hydrogen bonding. Similarly, the average structure from the simulation of the Wi-A complex with Survivin indicated a similar binding in the dimer-forming region. Further, Wi-A exhibited hydrogen bonding with the Thr5 residue of Survivin, similar to YM-155 (Figure 1C). The RMSD plot obtained for the protein–ligand complex revealed that Wi-A and YM-155 could interact stably with the Survivin protein (Figure 1D).

In addition to this, the interaction fraction diagram and the hydrogen occupancy plot of Wi-A indicated that the residues Phe101, Leu98, Glu94, Phe93, Thr5, and Leu6 formed many of the hydrogen bonds during the simulation (Figure 1E,F). These results were comparable to the interaction fraction diagram and hydrogen occupancy plot of YM-155 with Survivin. These data showed that Wi-A forms hydrogen bonds with six amino acid residues (Phe101, Leu98, Glu94, Phe93, Thr5, and Leu6). On the other hand, YM-155 forms hydrogen bonds with four amino acid residues (Leu98, Glu94, Thr5, and Leu6) (Figure 1G,H). Further, the binding energy calculation using the MM/GBSA method indicated that Wi-A could bind to Survivin strongly (binding energy of −54.68 kcal/mol) compared to YM-155 (binding energy of −44.38 kcal/mol). In contrast to Wi-A and YM-155, Wi-N did not show a stable interaction with Survivin, as evident from the RMSD plots of the simulations extended to 600 ns (Supplementary Figure S1B). The deviation observed from the RMSD plot, which is more than 3 Å, suggested that Wi-N is unstable in the binding pocket. The computational analyses revealed that Wi-A, but not Wi-N, has the potency to bind to the amino acids involved in Survivin homodimerization.

### 3.2. Downregulation of Survivin Protein and mRNA by Wi-A-Rich Extract of Ashwagandha Leaves (Wi-AREAL)

YM-155 has been shown to cause the transcriptional downregulation of Survivin. To this end, we examined its mRNA and protein levels in control and Wi-A-treated cells. The Survivin expression level was first determined in various human cancer cells compared to normal human fibroblasts. Based on this comparative analysis (Supplementary Figure S2B,C), we selected two cervical cancer cell lines (HeLa and ME-180) for further experiments.

**Figure 2:**
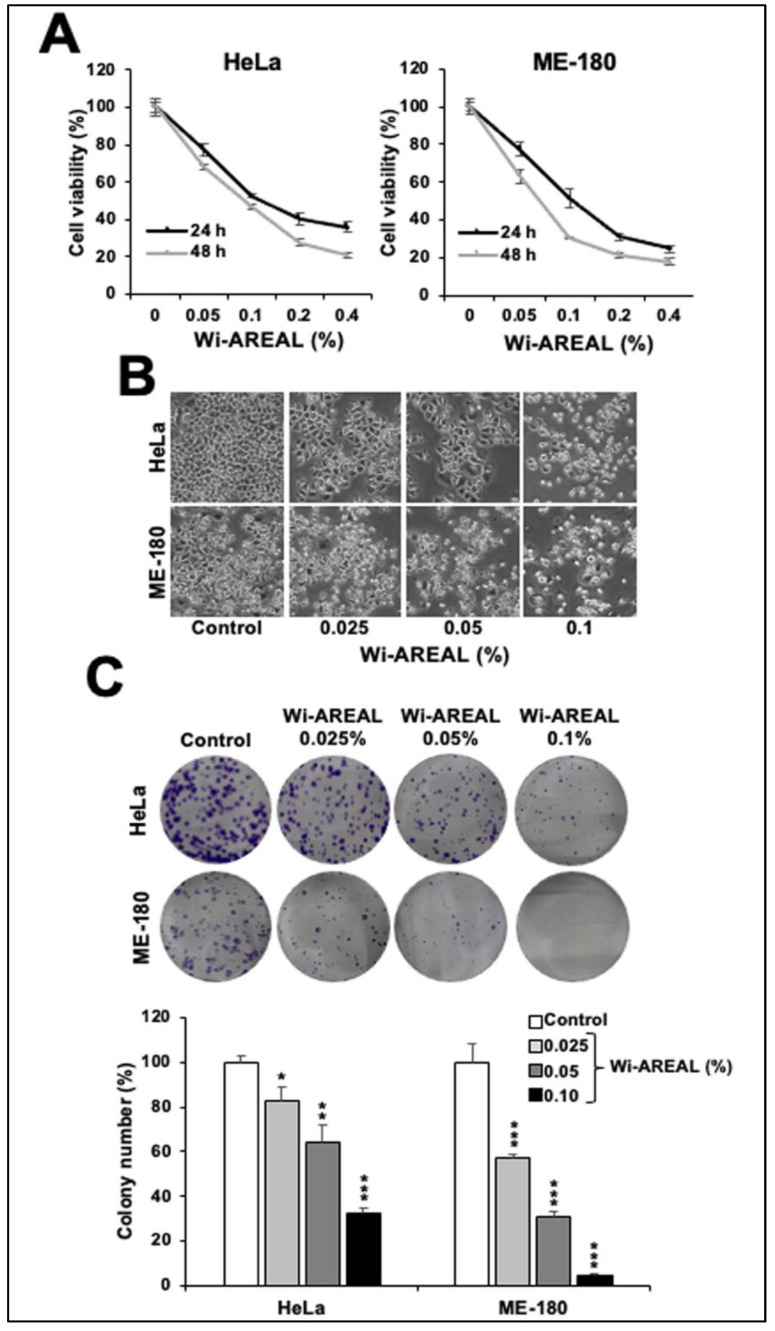
Effect of Wi-AREAL extract on the viability of cervical cancer cells. A dose-dependent decrease in viability (**A**) and increase in apoptotic cells (**B**) was observed in the short term (24–48 h treatment). (**C**) Long-term effects (10–15 days) showed a dose-dependent colony number and size reduction. Quantitation from three independent experiments is shown below (mean ± SD, n = 3), * *p* < 0.05, ** *p* < 0.01, *** *p* < 0.001 (Student’s *t*-test).

The dose-dependent cell viability analysis of Wi-AREAL-treated cells with respect to the control showed cytotoxicity in both cell lines, with an IC50 of 0.05–0.1% (Figure 2A). The morphological examination of cells revealed rounded and floating cells treated with these doses, signifying the occurrence of apoptosis. On the other hand, cells treated with lower doses (~0.025%) showed growth arrest-like morphology (Figure 2B). We also examined the long-term effect of Wi-AREAL by colony-forming assay.

Cells were subjected to Wi-AREAL (0.025%, 0.50%, and 0.1%) for ~10 h, followed by culture in normal medium for 10–15 days with regular medium changes on every third day. As shown in Figure 2C, Wi-AREAL-treated cultures of both cell lines showed a decrease in the colony number and the size of colonies. Consistent with the cell viability data, a dose-dependent decrease in colony formation was observed. HeLa cells showed a ~20, 40, and 70% reduction in colony number in cultures treated with 0.025, 0.0%, and 0.1% Wi-AREAL, respectively. Wi-AREAL-treated ME-180 cells showed a more substantial effect (~40, 70, and 95% reduction in colony number (Figure 2C)).

**Figure 3:**
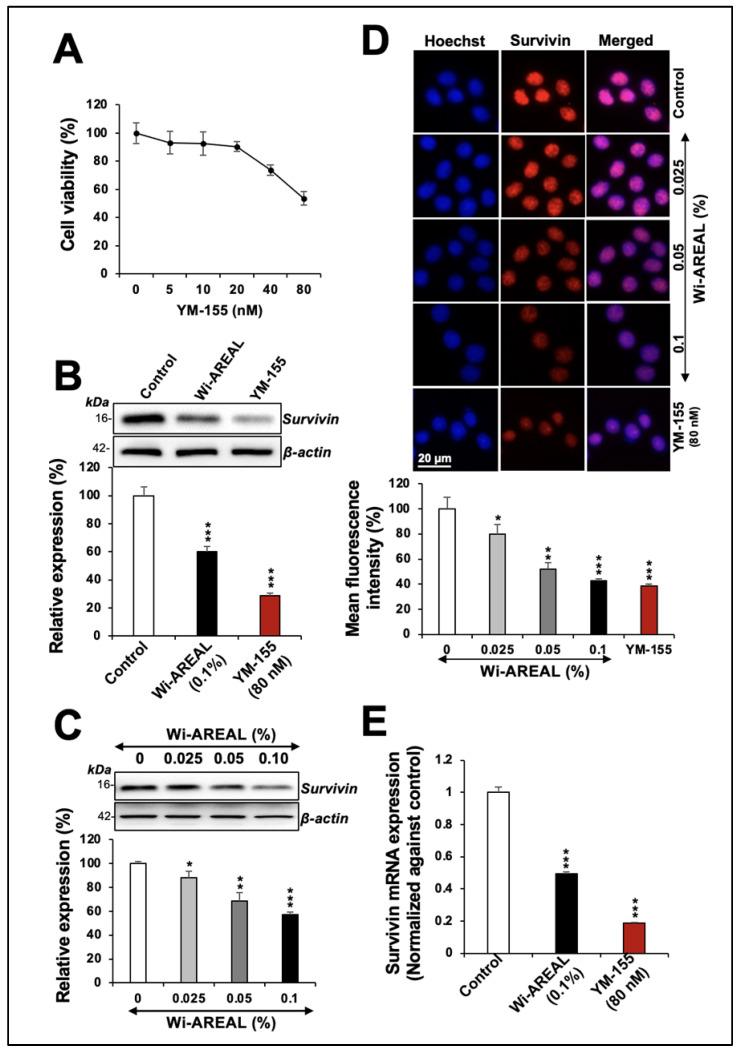
The effect of YM-155 and Wi-AREAL on Survivin protein and mRNA levels in HeLa cells. Dose-dependent effect of YM-155 on cell viability (**A**). Cells treated with YM-155 and Wi-AREAL showed a decrease in Survivin protein (**B**–**D**) as determined by Western blotting (**B**,**C**) and immunostaining (**D**). Quantitation from three independent experiments is shown below (mean ± SD, n = 3), * *p* < 0.05, ** *p* < 0.01, *** *p* < 0.001 (Student’s *t*-test). (**E**) Cells treated with YM-155 and Wi-AREAL showed a decrease in Survivin mRNA (mean ± SD, n = 3), *** *p* < 0.001 (Student’s *t*-test).

To investigate the involvement of Survivin in Wi-AREAL-induced cytotoxicity, we compared its effect with that of YM-155, an established inhibitor of Survivin that has been shown to downregulate its mRNA and protein expression. The dose-dependent viability assay showed a 20 and 50% decrease in cultures treated with 40 and 80 nM YM-155 (Figure 3A). Based on these data, 80 nM YM-155 was used parallel to 0.1% Wi-AREAL extract. HeLa cells treated with Wi-AREAL showed a reduction in Survivin protein (Figure 3B). Furthermore, an apparent dose-dependent decrease in Survivin was observed both in the Western blot (Figure 3C) and immunostaining (Figure 3D) assays. Notably, similar to the YM-155-treated cells, Wi-AREAL-treated cells showed a decrease in Survivin mRNA (Figure 3E), suggesting that Wi-AREAL may work similarly to YM-155.

### 3.3. Wi-AREAL-Treated Cells Showed G2 Growth Arrest and Apoptosis

**Figure 4:**
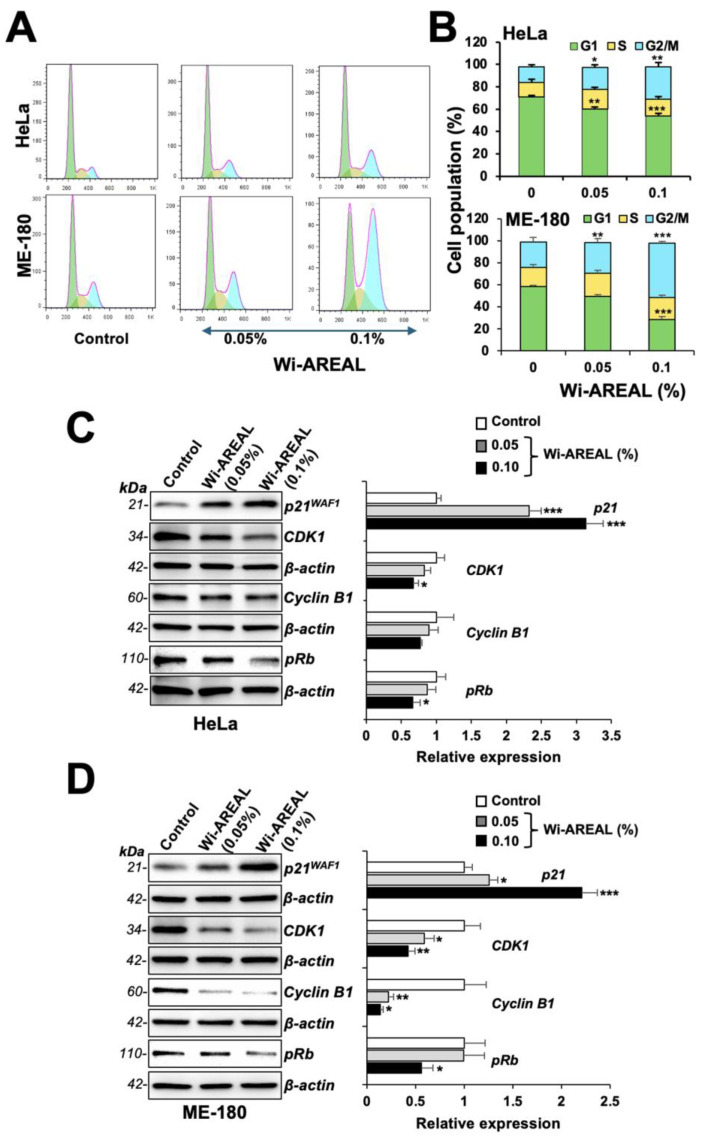
Cell cycle analysis of control and Wi-AREAL-treated HeLa and ME-180 cells. The treated cells exhibited G2-M arrest (**A**,**B**), increased p21WAF1, and decreased CDK1, cyclinB1, and pRb (**C**,**D**) levels in both cell lines. Quantitation from three independent experiments is shown on the right (mean ± SD, n = 3), * *p* < 0.05, ** *p* < 0.01, *** *p* < 0.001 (Student’s *t*-test).

Next, we performed a cell cycle analysis of the control and Wi-AREAL (0.05 and 0.1%) -treated cells. As shown in Figure 4A,B, the treated cells showed an increase in cell population in the G2-M phase consistent with the reduction in Survivin, as shown in Figure 3. Of note, a dose-dependent increase in the G2-M population was recorded in both cell lines. Furthermore, 0.1% Wi-AREAL-treated ME-180 cells showed a higher number of cells arrested in the G2-M phase as compared to HeLa cells, and this was consistent with the more substantial reduction in colony number in ME-180 shown in Figure 2C. The biochemical analysis of molecular markers involved in cell cycle progression revealed a dose-dependent increase in p21^WAF1^ and a decrease in CDK1, cyclin B1, and pRB proteins in cells treated with Wi-AREAL. The data were consistent with G2-M arrest in both cell lines and showed a more substantial effect on ME-180 than on HeLa cells (Figure 4C,D).

**Figure 5:**
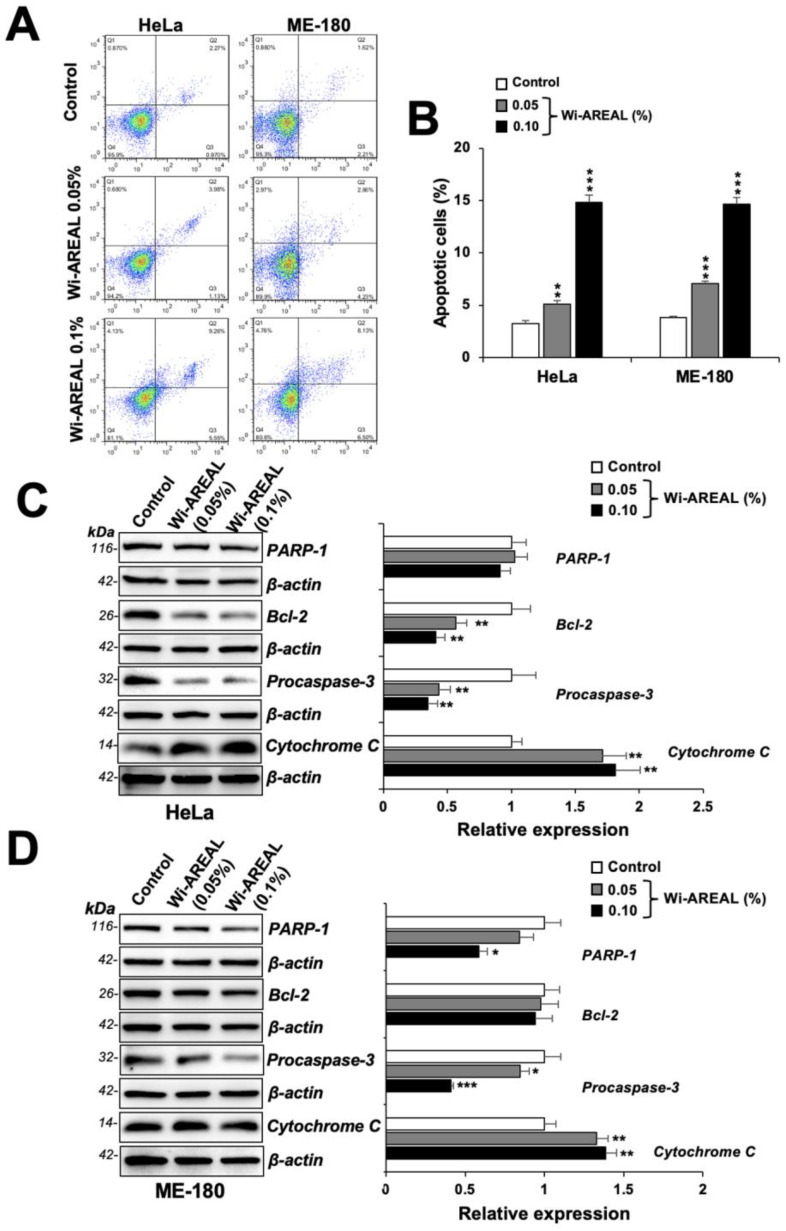
Flow cytometry analyses of control and Wi-AREAL-treated cells. The treated cells showed a dose-dependent increase in apoptotic cells (**A**,**B**) and Cytochrome C (**C**,**D**) and a decrease in PARP-1, Bcl2, and procaspase (**C**,**D**) in both cell lines. Quantitation from three independent experiments is shown on the right (mean ± SD, n = 3), * *p* < 0.05, ** *p* < 0.01, *** *p* < 0.001 (Student’s *t*-test).

Microscopic observations of control and Wi-AREAL-treated cells of both cell types showed a rounded apoptotic morphology, as shown in Figure 2B. Hence, we next performed flow cytometry analysis for apoptosis. As shown in Figure 5A,B, both cell lines showed a dose-dependent increase in apoptotic cells upon treatment with Wi-AREAL (0.05 and 0.1%). Furthermore, biochemical analyses of proteins involved in apoptosis revealed a dose-dependent decrease in procaspase-3 and an increase in Cytochrome C in both cell lines (Figure 5C,D). Wi-AREAL-treated HeLa and ME-180 cells showed no significant change in PARP-1 and Bcl-2, respectively. Taken together, these data demonstrated the occurrence of growth arrest and apoptosis in Wi-AREAL-treated cancer cells.

### 3.4. Low Nontoxic Doses of Wi-AREAL Showed Anti-Metastasis Potential

We next hypothesized that Wi-AREAL may inhibit the metastasis of cancer cells and performed in vitro assays (migration, invasion) in control and treated ME-180 cultures. A low nontoxic concentration of Wi-AREAL (0.01%) was used along with 0.5 μM Wi-A and 40 nM YM-155 as positive controls, as reported in earlier studies [21,22,23,24,25,26,27,32,33,36].

**Figure 6:**
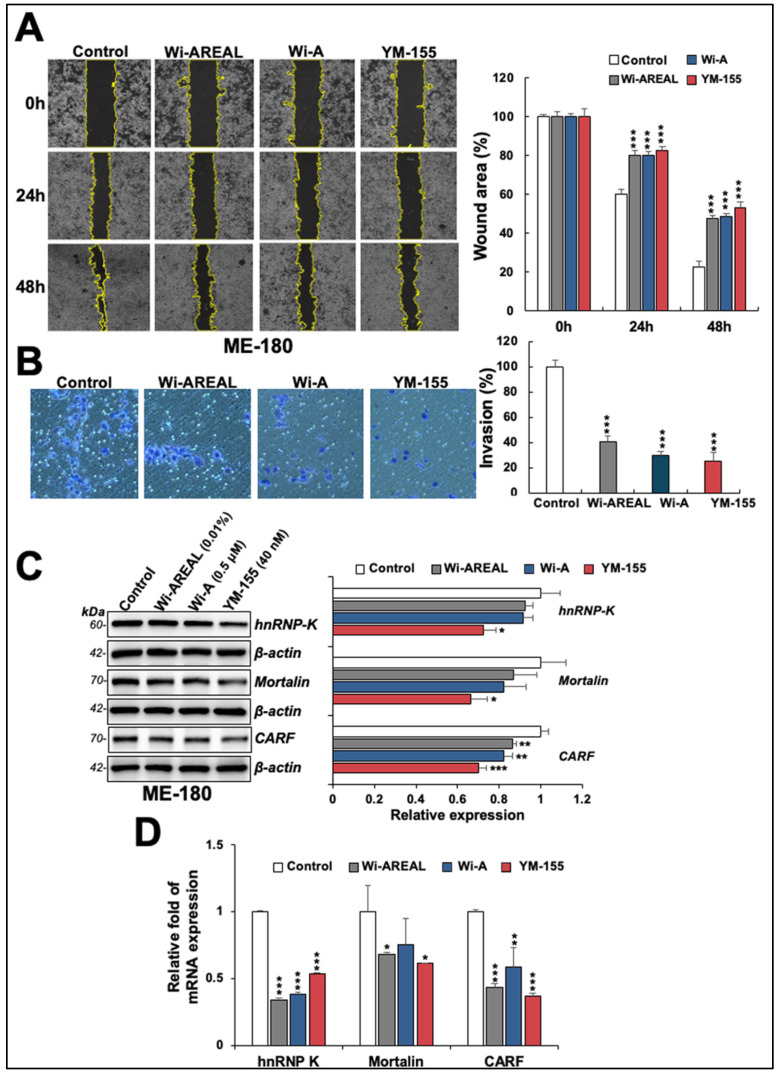
Effect of a low dose of Wi-AREAL on cell migration and invasion characteristics. ME-180 cells treated with Wi-AREAL showed a decrease in cell migration (**A**) and invasion (**B**) comparable to that in cells treated with either Wi-A or YM-155. Quantitation from three independent experiments is shown on the right (mean ± SD, n = 3), *** *p* < 0.001 (Student’s *t*-test). The treated cells showed decreased hnRNP-K, mortalin, CARF proteins (**C**), and mRNA (**D**) (mean ± SD, n = 3), * *p* < 0.05, ** *p* < 0.01, *** *p* < 0.001 (Student’s *t*-test).

As shown in Figure 6A, a decrease in the migration ability of HeLa cells treated with Wi-AREAL was detected compared to the control cells in wound scratch assays. We found that the effect of 0.01% Wi-AREAL was comparable with that of Wi-A (0.5 μM) and YM-155 (40 nM). The cell invasion assay also revealed a decrease in Wi-AREAL-treated cells that was similar to that seen for Wi-A and YM-155 (Figure 6B). The analysis of molecular markers of cell migration and metastasis (hnRNP-K, mortalin, and CARF) also showed a change in treated cells. YM-155 caused a substantial and statistically significant decrease in all three proteins. Wi-AREAL- and Wi-A-treated cells showed a significant reduction in CARF but not in hnRNP-K or mortalin proteins (Figure 6C). We extended the analysis to transcriptional regulation. qRT-PCR analysis in control and treated cells showed a remarkable reduction in these mRNAs in the latter, and the results were quantitatively similar to those for Wi-A- or YM-155-treated cells (Figure 6D).

**Figure 7:**
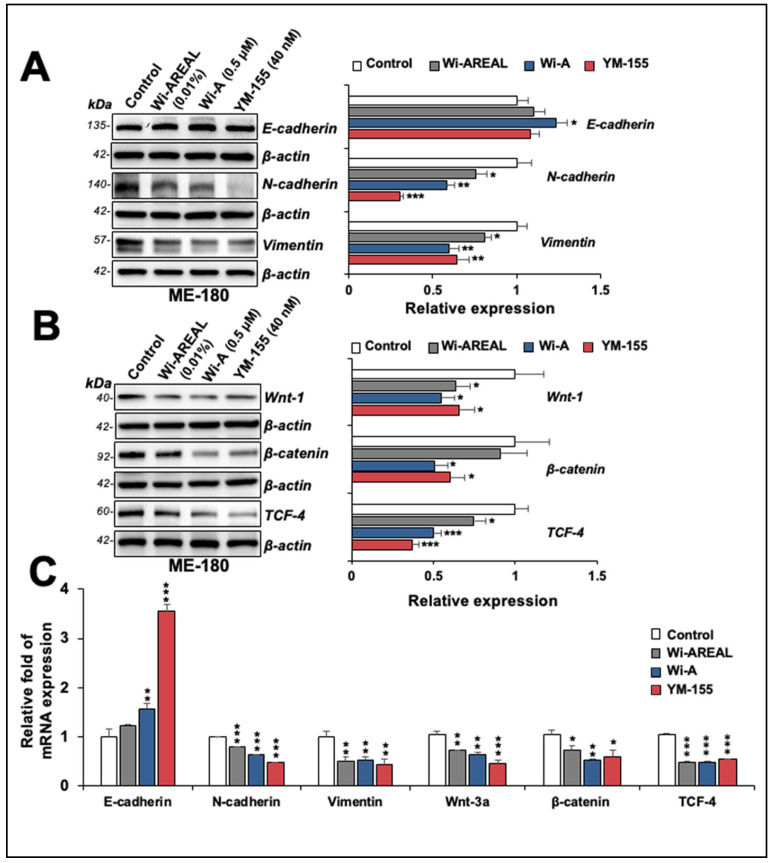
Effect of a low dose of Wi-AREAL on EMT (epithelial–mesenchymal transition). The treated cells showed reduced N-cadherin, Vimentin, Wnt-1, β-catenin, and TCF-4 at the protein (**A**,**B**) and mRNA (**C**) levels. (mean ± SD, n = 3), * *p* < 0.05, ** *p* < 0.01, *** *p* < 0.001 (Student’s *t*-test).

We also examined the expression of proteins involved in EMT in control and Wi-AREAL (0.01%) -treated HeLa cells. Consistent with the decrease in migration and invasion potential of Wi-AREAL-treated cells, as shown in Figure 6, we found an increase in E-cadherin (Figure 7A). Wi-A-treated cells showed a significant increase; Wi-AREAL- and YM-155-treated cells showed a minor and inconsistent increase. On the other hand, N-cadherin and Vimentin showed a significant decrease (Figure 7A) in cells treated with either of the three (Wi-AREAL, Wi-A, and YM-155) groups. The analysis of Wnt-1, β-catenin, and TCF-4 also showed a reduction in treated vs. control cells (Figure 7B). Of note, the effect of Wi-AREAL on Wnt-1 and TCF-4 proteins was quantitatively similar to that of the two positive controls, Wi-A and YM-155. On the other hand, Wi-AREAL-treated cells showed a small and insignificant decrease in β-catenin. The effect was also detected at the transcript level. As shown in Figure 7C, YM-155- and Wi-A-treated cells showed a significant increase in E-cadherin mRNA. Wi-AREAL-treated cells showed a small and insignificant increase. These results were consistent with the protein expression data shown in Figure 7A. On the other hand, a decrease in N-cadherin, Vimentin, Wnt-3a, β-catenin, and TCF-4 in Wi-AREAL-treated cells was quantitatively matched to that in the Wi-A and YM-155 positive controls (Figure 7C). Taken together, these data demonstrated that Wi-AREAL caused a reduction in the migration and invasion characteristics of cancer cells through the inhibition of EMT signaling, similar to its purified active component (Wi-A), as well as YM-155, an established Survivin inhibitor.

## 4. Discussion

Survivin protein plays a crucial role in regulating cell proliferation. It controls mitosis and apoptotic signaling through independent as well as interconnected pathways. Previous studies have shown that it binds to and inhibits proteins involved in intrinsic (Bcl-2, Bcl-xl, BAX, Mcl-1, caspase-9) and extrinsic (TRAIL, FAS, FLIP, caspase-3, caspase-7) apoptotic pathways. Additionally, Survivin interacts with other regulators of cell division and apoptosis mediators. For example, an increase in Survivin in cancer cells leads to the upregulation of various signaling pathways, including phosphatidylinositol 3-kinase (PI3K/AKT), Wnt/β-catenin, EMT, VEGF, and other regulators of metastasis and angiogenesis [1,2,53,54,55]. Wild-type p53 was seen to repress Survivin at both the mRNA and protein levels. On the other hand, the overexpression of Survivin led to the prevention of p53-induced cell death in a dose-dependent manner, suggesting that these two crucial cell growth and cell death controllers work together to regulate these processes [56]. The overexpression of Survivin has also been clinically connected to poor prognosis and therapeutic resistance. Due to the essential role of Survivin in cell division, its multi-functionality, and its frequent upregulation in various cancers, it is considered a viable therapeutic target. Furthermore, its expression is very low in normal cells, offering drug selectivity toward the cancer cells [54].

The induction of apoptosis is one of the most eminent cellular mechanisms to evade cancer. On the onset of apoptotic signaling, mitochondria release SMAC (small mitochondrial activator of caspase), which binds to the BIR domain of IAPs (XIAP, cIAP1, cIAP2, and Survivin), neutralizing their apoptosis-inhibitory activity [1,57]. SMAC mimetics, including small molecules (such as PZ-6-QN, LCL161, and TL32711) and peptides that can bind to BIR domains of IAPs, have thus been predicted to possess cancer therapeutic activity [40,54]. Several other small-molecule inhibitors of Survivin have been reported and are being tested in preclinical and clinical studies [54]. These include (a) transcriptional repressors (YM-155 and EM-1421), (b) translational repressors (oligonucleotides: EZN-3042 and LY2181308, and ribozymes: CUA110 and GUC294), and (c) structural regulators controlling the dimerization/stability of the protein (LQZ-7F, AICAR, LLP3, and LLP9) [54]. These molecules modulate Survivin expression in multiple ways, including (i) direct interactions with genes, mRNA, or protein; (ii) indirect inhibition by binding to proteins that interact with Survivin and regulating its stability; and (iii) the enhancement of its degradation [18,20,53,54,57].

**Figure 8:**
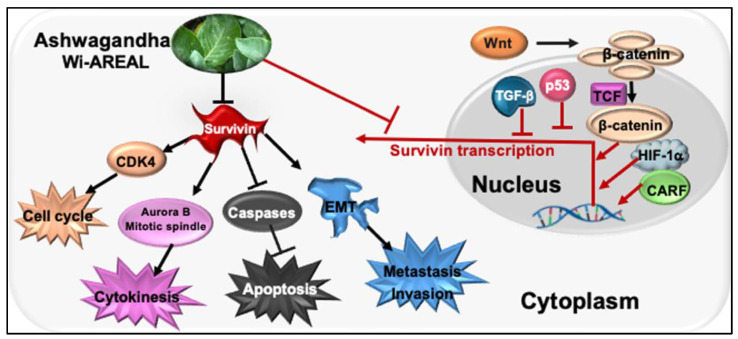
The schematic model illustrates the impact of Wi-REAL on cancer cell characteristics through the inhibition of Survivin signaling.

An earlier molecular docking study predicted the binding potential of Wi-N to the BIR5 domain [39]. Since the homodimerization of Survivin is critical for its function, we determined the docking capability of Wi-A and Wi-N to this domain of Survivin in reference to its established inhibitor YM-155, which works both at the mRNA and protein levels [54,58]. YM-155 was shown to be cytotoxic to neuroblastoma cells and their multi-drug-resistant clones (IC50 ranging from 0.49~49.3 nM) and targeted Survivin specifically, as validated by RNAi-mediated Survivin depletion assays [58]. In light of these data, we chose YM-155 as a positive control in this study to investigate the effect of Wi-AREAL on Survivin. Furthermore, the YM-155 response was shown to be dependent on the wild-type p53 function [56,58]. Of note, Wi-A has earlier been shown to activate and restore wild-type p53 function in cancer cells [31,32,33,36,59,60]. Hence, from this viewpoint, the simultaneous targeting of Survivin and p53 by Wi-A may be suggested as a proper poly-molecular therapeutic strategy, as shown in Figure 8.

Molecular docking analysis revealed that Wi-A, but not Wi-N, can block Survivin dimerization. The binding characteristics of Wi-A to Survivin showed stability and substantial similarity with YM-155, an established inhibitor of Survivin. In several earlier studies, Wi-A has been shown to possess stronger anticancer activity as compared to Wi-N [29,30,31,32,36,37,61,62], which may be explained by the differential action of these closely related withanolides on Survivin dimerization.

Survivin is considered a powerful, unique target for cancer therapy due to its well-established multiple functions in regulating the cell division, apoptosis, stress, stemness, and drug response of cancer cells [63,64,65]. Wi-A, on the other hand, has recently been shown to possess anti-inflammatory, antiherpetic, antifibrotic, immunosuppressive, and anticancer potentials that operate through multiple targets and molecular pathways [31,32,33,36,59,66,67]. We used Wi-A and Wi-AREAL and demonstrated their Survivin-targeting activity, promoting apoptosis and inhibiting metastatic properties in cancer cells through multiple pathways (Figure 8), suggesting their place in the expanding list of molecular inhibitors of Survivin [68].

## 5. Conclusions

Through molecular docking approaches, we discovered that Wi-A, but not Wi-N, may block Survivin dimerization similarly to YM-155 (an established inhibitor of Survivin).

Cervical cancer cell lines treated with Wi-A-rich extract from Ashwagandha leaves (Wi-AREAL) showed active growth arrest and/or apoptosis at high (0.05~0.1%) and the inhibition of metastatic characteristics at low (0.01%) concentrations, supported by biochemical markers of these phenotypes. The study sheds light on the molecular basis of Wi-A’s stronger anticancer activity compared to Wi-N. It proposes Wi-AREAL as a natural inhibitor of Survivin with drug-like properties that could benefit cancer treatment.

## Data Availability

The manuscript and Supplementary Information files contain all datasets used and/or analyzed during the current study.

## Supplementary Materials

The following supporting information can be downloaded at: https://www.mdpi.com/article/10.3390/cancers16173090/s1, Figure S1. (A) Structures of Withaferin A (Wi-A), Withanone (Wi-N) and YM-155. (B) Docking pose of Withanone (Wi-N) to Survivin along with its RMSD plot showing deviation indicating an unstable interaction after simulation of 600 ns. Figure S2. HPLC profile of Wi-AREAL. The amounts of Withaferin-A (Wi-A) and Withanone (Wi-N) in reference to the standard compounds are shown in (A). Additionally, a Western blot displaying the expression level of the Survivin protein in various human normal and cancer cells (B) and four cervical cancer cell lines (C) is included. Table S1. List of primary antibodies used for Western blotting and immunostaining. Table S2. Primer sequences used for qRT-PCR. References [69,70] are cited in the Supplementary Materials.
