# Supplementary material for: Molecular Insights into the Anticancer Activity of Withaferin-A: The Inhibition of Survivin Signaling
Source: Cancers (Basel). 2024 Sep 5;16(17):3090. doi: 10.3390/cancers16173090 (PMC11394585; doi:10.3390/cancers16173090)
Supplement: Supplementary file 1 [file cancers-16-03090-s001.zip › cancers-3114994-supplementary.pdf]

SUPPLEMENTARY INFORMATION 1

Molecular Insights into the Anticancer Activity of Withaferin-A: The Inhibition of Survivin Signaling

Renu Wadhwa [1,†], Jia Wang [1,†], Seyad Shefrin [2], Huayue Zhang [1], Durai Sundar [2,3,*] and Sunil C. Kaul [1,*]

[1] AIST-INDIA DAILAB, National Institute of Advanced Industrial Science & Technology (AIST), Central 4-1, Tsukuba 305-8565, Japan; renu-wadhwa@aist.go.jp (R.W.); wangjia@szbl.ac.cn (J.W.); s2130297@u.tsukuba.ac.jp (H.Z.)
[2] Department of Biochemical Engineering & Biotechnology, Indian Institute of Technology (IIT) Delhi, Hauz Khas, New Delhi 110 016, India; bez188440@dbeb.iitd.ac.in
[3] Institute of Bioinformatics and Applied Biotechnology (IBAB), Bengaluru 560 100, India
* Correspondence: sundar@dbeb.iitd.ac.in (D.S.); s-kaul@aist.go.jp (S.C.K.)
† These authors contributed equally to this work.

Supplementary Figure S1. (A) Structures of Withaferin A (Wi-A), Withanone (Wi-N) and YM-155. **(B)** Docking pose of Withanone (Wi-N) to Survivin along with its RMSD plot showing deviation indicating an unstable interaction after simulation of 600 ns.

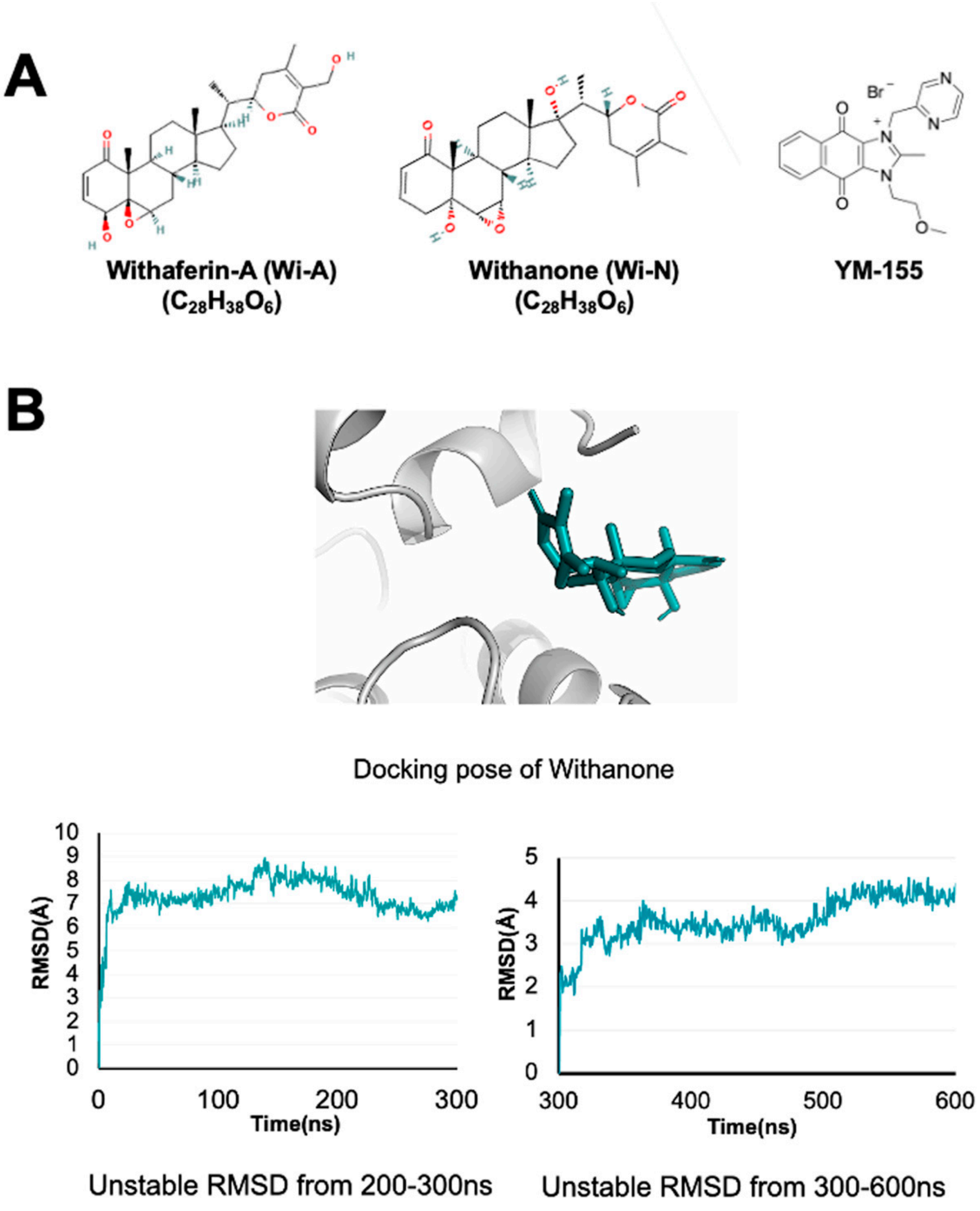

Supplementary Figure S2. HPLC profile of Wi-AREAL. The amounts of Withaferin-A (Wi-A) and Withanone (Wi-N) in reference to the standard compounds are shown in (**A**). Additionally, a Western blot displaying the expression level of the Survivin protein in various human normal and cancer cells (**B)** and four cervical cancer cell lines (**C**) is included.

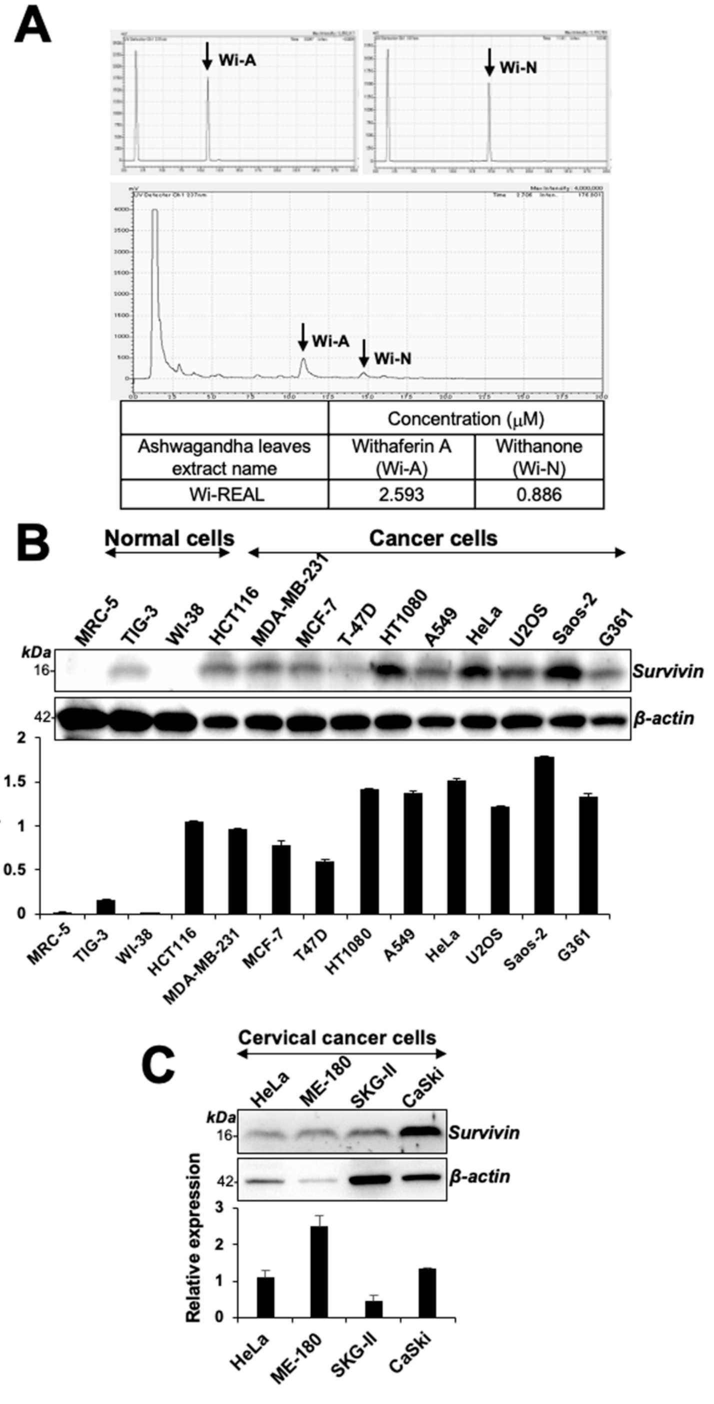

Supplementary Table S1. List of primary antibodies used for Western blotting and immunostaining.

Antibody	Catalog No.	Source
Survivin	71G4B7	Cell Signaling Technology, Danvers, MA, USA
p21WAF1	12D1	
pRb	9301S	
hnRNP-K	R332	
E-cadherin	4A2	
N-cadherin	D4R1H	
CDK1	610037	BD Biosciences, San Jose, CA, USA
Cyclin B1	GNS1	Santa Cruz Biotechnology, CA, USA
PARP-1	F-2	
Procaspase 3	E-8	
Vimentin	V9	
Wnt-1	E-10	
β-catenin	E-5	
TCF-4	D-4	
Bcl-2	ab196495	Abcam, Cambridge, UK
Cytochrome C	ab133504	
β-actin	643807	BioLegend, Tokyo, Japan
CARF	A-10	Raised in our lab [1]
Mortalin	37-6	Raised in our lab [2]

1. Kalra, R.S.; Chaudhary, A.; Omar, A.; Li, X.; Khurana, M.; Kaul, S.C.; Wadhwa, R. Stress-induced changes in CARF expression serve as a quantitative predictive measure of cell proliferation fate. *Exp Cell Res* **2023**, *429*, 113669, doi:10.1016/j.yexcr.2023.113669.
2. Shiota, M.; Ikeda, Y.; Kaul, Z.; Itadani, J.; Kaul, S.C.; Wadhwa, R. Internalizing antibody-based targeted gene delivery for human cancer cells. *Hum Gene Ther* **2007**, *18*, 1153-1160, doi:10.1089/hum.2007.087.

Supplementary Table S2. Primer sequences used for qRT-PCR.

**Gene (human)**	**Sequence (5′-3′)**
Survivin forward	CCACTGAGAACGAGCCAGACTT
Survivin reverse	GTATTACAGGCGTAAGCCACCG
HNRNPK forward	GCAGATGGCTTATGAACCACAGG
HNRNPK reverse	AATCCGCTGACCACCTTTGCCA
Mortalin forward	AGCTGGAATGGCCTTAGTCAT
Mortalin reverse	CAGGAGTTGGTAGTACCCAAATC
CARF forward	TCAAAGTGACAGATGCTCCA
CARF reverse	CGTTGAACTGTTTTCCTGCT
E-cadherin forward	CGGGAATGCAGTTGAGGATC
E-cadherin reverse	AGGATGGTGTAAGCGATGGC
N-cadherin forward	CCTCCAGAGTTTACTGCCATGAC
N-cadherin reverse	GTAGGATCTCCGCCACTGATTC
Vimentin Forward	CCTTGAACGCAAAGTGGAATC
Vimentin Reverse	GACATGCTGTTCCTGAATCTGAG
Wnt-3α Forward	CAAGATTGGCATCCAGGAGT
Wnt-3α Reverse	TCCCTGGTAGCTTTGTCCAG
β-catenin Forward	TGATGGAGTTGGACATGGCCATGG
β-catenin Reverse	CAGACACCATCTGAGGAGAACGCA
TCF4 Forward	GCCTCTTCACAGTAGTGCCATG
TCF4 Reverse	GCTGGTTTGGAGGAAGGATAGC
18S forward	CAGGGTTCGATTCCGTAGAG
18S reverse	CCTCCAGTGGATCCTCGTTA
